# Supplementary material for: Are carbon emissions trading and green financial instruments synergistic? -Comprehensive quantitative research based on content analysis
Source: PLoS One. 2024 Mar 7;19(3):e0298601. doi: 10.1371/journal.pone.0298601 (PMC10919601; doi:10.1371/journal.pone.0298601)
Supplement: S1 Data — (ZIP) [file pone.0298601.s001.zip › Data/地级市Z_F工作报告.zip!/╦─┤¿/┤∩╓▌╩╨/└┤╫╘╣1⁄2╓┌║┼í╛┬φ┐╦╩2╛▌═°í┐_2014.docx]

各位代表：

现在，我代表市人民政府向大会报告工作，请予审查，并请市政协各位委员提出意见。

一、2013年工作回顾

过去一年，面对复杂严峻的形势，在省委、省政府和市委的坚强领导下，全市上下认真落实省委“三大发展战略”，扎实推进“三大总体战略工程”，迎难奋进、拼搏实干，经济发展稳中有进、稳中向好，社会保持和谐稳定。全市实现地区生产总值1245.4亿元，增长10.2%。地方公共财政收入60.3亿元，增长15.9%。全社会固定资产投资1002.9亿元，增长21.4%。社会消费品零售总额493.4亿元，增长14.8%。城镇居民人均可支配收入18915元，农民人均纯收入8001元，分别增长11.6%和13.5%。城镇登记失业率4.1%。人口自然增长率4.42‰。居民消费价格上涨2.5%。节能减排省控目标全面完成。

一年来，我们主要做了以下工作：

（一）调整优化产业结构，经济保持持续健康发展。三次产业结构调整为21.4:53.1:25.5。工业强市战略深入实施。规模以上工业增加值338.1亿元，增长10.1%，新培育十亿级企业4户、亿元企业16户，新增规模以上工业企业53户。完成产业园区“七通一平”5800亩，建设标准厂房35万平方米，开工建设亿元以上产业项目47个。达州经开区纳入全省千亿园区规划，大竹、宣汉产业园区纳入全省500亿园区规划。新增市级以上企业技术中心7家，创建高新技术企业6家，新获授权专利417件。整顿关闭煤矿企业71户，淘汰落后产能企业12户。服务业加快发展。实现增加值317.1亿元，增长10.4%，增速首次超过地区生产总值和规模以上工业增加值增速。达州跻身全国二级物流园区布局城市和全省首批现代物流试点城市。新增金融机构9家，金融业实现增加值33亿元。旅游特色化和标准化建设成效显著，实现旅游总收入75.2亿元。现代农业稳步推进。粮食生产实现“七连增”，总产达到281.7万吨。新建特色产业基地14.5万亩、畜牧养殖小区316个，“六带三区两基地”农业产业发展带初步形成。新培育市级以上龙头企业17家，新发展农民专业合作组织298家，直接带动77万农户增收，广袤农村正成为全面建成小康社会的全新动力源。

（二）突出抓好项目建设，跨越发展基础更加牢固。深入开展“项目突破年”活动，编制储备次级突破重点项目900个，加快实施重点项目150个，集中攻坚重大工程项目25个，完成投资337亿元。巴达高速公路、南大梁高速公路南充至渠县段建成通车，实现县县通高速公路；达州机场新开通达州至昆明航线，机场异地扩迁选址获得批准；达州火车站扩能改造竣工投入使用，达巴铁路、火车货站南迁等项目顺利实施；环城公路二期、金南大桥、达宣达开快速通道开工建设，建成通乡（村）公路2870公里。“3+10+1”骨干水利工程扎实推进，白岩滩等3座中型水库完成投资突破6亿元，病险水库除险加固45座，解决31万农村人口饮水安全问题。中青家居、一新机电等商贸物流项目竣工运营；红星美凯龙等“十亿级”商贸物流项目进展顺利。川投天然气发电、西部皮鞋城等项目即将投产，安凯汽车、颜锋铝业等项目加快建设。着力强化资金、土地等要素保障，全市筹集项目前期工作经费超过1亿元，社会融资突破230亿元，土地整理新增耕地2.6万亩，实现土地出让收入65.9亿元，均实现新突破。

（三）加快推进城镇建设，城乡环境面貌明显改观。坚持“四层架构”，注重规划引领，强化主城带动，全市城镇化率达到37.8%，提高1.7个百分点。达县撤县设区和达州经开区扩区调位全面完成，北城滨江、南城三里坪、西城莲花湖和马踏洞片区建设加快推进，“三横五纵一环五射”城区骨干路网加速构建，中心城区建成区面积达到68平方公里、人口达到82.5万。加快推进县域经济发展和小城镇建设，地区生产总值超200亿元县达到2个，纳入全省“百镇建设试点镇”7个，宣汉县成功纳入全省少数民族地区待遇县。坚持产村相融成片建设幸福美丽新村，一批新村聚居点建成入住。推进公交优先发展，中心城区更新公交车辆189辆，实现GPS定位和公交智能刷卡乘车。完成火车站公交首末站和社会停车场建设，火车站交通秩序得到明显改善。开展城乡环境综合治理，改造背街小巷122条，打造特色街道17条，城区广告整治、治尘治噪、风貌塑造初见成效。加大城乡绿化力度，完成营造林30万亩，新增城镇绿地39万平方米，森林覆盖率达到41%。加快城市环保设施建设，新建雨污分流管网42公里，城镇污水、垃圾处理率分别达45%、100%。顺利通过省级森林城市和文明城市验收。

（四）着力办好民生实事，人民生活水平稳步提升。民生工程扎实推进。各项民生投入180.7亿元，占公共财政支出的70.6%。新增城镇就业3.7万人，零就业家庭多年保持动态清零。城乡居民养老、医疗保险实现制度全覆盖，重特大疾病医疗救助和高龄老人生活补助制度更加完善，城乡低保人数达到50.9万人，“五保”集中供养率提高到46%。完成棚户区及农村危房改造2万余户，建成保障性住房1.15万套。实施扶贫整村推进和连片扶贫开发项目78个，帮助8.7万农村群众脱贫。社会事业加快发展。新改扩建中小学校105所、公办幼儿园43所，西南职教园区加快建设。编制实施中心城区教育资源布局规划，城区学校“大班额”问题逐步缓解。市图书馆、展演基地等公共文化设施加快建设，成功举办第三届全国新农村文化艺术展演。实施城乡医疗卫生服务项目328个，县级公立医院全面取消药品加成。全民健身活动广泛开展，成功举办首届市职工运动会和农民运动会。计生惠民政策全面落实。第三次全国经济普查顺利推进。“平安达州”建设深入实施，安全生产和食品药品安全监管切实加强。

（五）深入推进改革开放，体制机制活力不断增强。国企改革取得新突破，筹集改革资金1.2亿元，成功争取核销养老保险挂账3.3亿元，基本完成达棉总厂、川鼓公司等28户国企改革任务。国有资本经营预算全面推行，市县公共资源交易平台不断完善，政府性公共资源管理得到加强。民营经济加快发展，私营企业、个体工商户分别达到1.6万户、11万户，民营经济增加值占地区生产总值比重提高到59.1%。事业单位改革、文化体制改革、行政机构改革有序推进，“营改增”试点顺利实施。城市规划、建设、管理职能实现调整优化，市、区管理体制进一步明晰。招商引资取得新成效，签约10亿元以上项目12个、亿元以上项目137个，引进到位资金564亿元。完成进出口总额2.5亿美元，出口突破1.3亿美元。区域合作不断深化，成功举办第四届秦巴地区商品交易会。

（六）切实转变工作作风，政府自身建设得到加强。自觉接受人大法律监督、工作监督和政协民主监督，认真办理人大代表建议、批评和意见115件，政协委员提案352件。行政执法自由裁量权得到细化量化，行政权力依法规范公开运行平台全面建立。政务服务体系不断健全，“两集中、两到位”有效落实。取消调整73项行政审批项目和13项公共服务事项，行政审批事项进入全省最少市州行列。政府信息公开和新闻发布制度建立完善，重大行政决策听证全面开展。市应急联动指挥中心建成运行，应急救援能力不断提升。政府性投资项目评审和审计监督切实加强，政府性债务审计如期完成。认真执行“改进工作作风密切联系群众”有关规定，行政执法队伍作风整顿取得阶段性成效。廉政建设和反腐败工作扎实推进，查处了一批违纪违法案件。

各位代表，2013年全市各条战线都争先创优，奋发有为。国防动员、人民防空、民兵预备役建设、双拥共建和优抚安置工作不断加强，军政军民关系进一步密切。妇女儿童、青年、慈善、老龄、残疾人事业持续发展，民族、宗教、对台、外事、侨务、统计、档案、保密、气象和地方志等工作取得新进步。

过去一年的成绩来之不易，离不开省委、省政府的高度重视、亲切关怀，离不开市委的正确领导、科学决策，离不开市人大、市政协的有效监督、大力支持，离不开全市人民的团结奋斗、辛勤付出。在此，我代表市人民政府，向全市人民，向人大代表、政协委员，向各民主党派、工商联、无党派人士和人民团体，向驻达解放军、武警官兵和政法干警，向所有关心支持达州发展的社会各界人士表示衷心的感谢！

我们也清醒地看到，在发展和工作中还存在不少困难和问题。经济发展中长期累积的结构性矛盾与宏观环境不利因素叠加，影响了去年全市经济部分预期目标的实现。特别是规模以上工业发展不快、质量不高，现代农业和服务业比重偏低，转型升级发展面临较大压力。项目建设后劲乏力，民间投资意愿不强，争取上级支持、推进招商引资尚需加大力度。城乡居民增收缺乏有力支撑，社会不和谐不稳定因素仍然较多，保障和改善民生任务艰巨。政府公共服务和依法治理能力还需提升，作风不实、服务不优、效能不高、为政不廉等问题仍不同程度存在。我们一定高度重视人民群众的关切，勇于直面自身工作中的问题，采取有力措施切实加以解决，绝不辜负全市人民的期望。

二、2014年主要目标任务

2014年是在新的起点上全面深化改革的开局之年，也是实施“十二五”规划的关键之年。今年政府工作的总体要求是：认真贯彻党的十八大和十八届二中、三中全会精神，全面落实中央和省、市委经济工作会议部署，以改革统领经济社会发展全局，坚持科学发展、加快发展、追赶跨越的工作基调，深入实施“三大总体战略工程”，充分释放改革动力，挖掘经济增长潜力，保持转型升级定力，全面激发市场活力，着力保障和改善民生，促进全市经济持续健康发展、社会和谐稳定，努力在全省多点多极支撑发展战略中率先实现次级突破，加快建设川渝鄂陕结合部区域中心城市和川东北经济区核心增长极。

今年经济社会发展的主要预期目标是：地区生产总值增长11%；全社会固定资产投资增长15%；社会消费品零售总额增长13%；地方公共财政收入增长12%；城镇居民人均可支配收入增长12%，农民人均纯收入增长13%；居民消费价格涨幅控制在3.5%左右；城镇登记失业率控制在4.1%以内；人口自然增长率控制在5‰以内；完成省政府下达的节能减排任务。

围绕上述总体要求和预期目标，今年政府工作要着重把握好以下四个方面：

（一）更加注重做大总量与提升质量相统一。发展仍是解决我市所有问题的关键，转型是我们必须迈过的坎。顺应我市经济发展的阶段性特征，坚持把加快发展作为最紧迫的任务，全面落实省委“三大发展战略”，深入实施市域经济“842”工程，大力上项目扩投资，全力兴产业强企业，着力抓城镇带乡村，努力培育经济增长新亮点，切实保持较快的经济增速，争取在千亿市州中走在前列，推动率先次级突破。坚持以发展促升级、以增量调结构，大力推进产业结构、产品结构、投资结构转型升级，促进一产扩量降比夯基础、二产扩量提质调结构、三产扩量提比强功能，积极争创达州优势，实现持续健康发展。

（二）更加注重深化改革与创新管理相驱动。坚持向改革要速度，深入研究和对接国家和省改革部署，逐项细化改革实施方案，稳步推进经济体制、文化体制、社会体制、生态文明体制等各领域改革，进一步理顺市区体制、明晰部门职责，以改革的思路和办法破解制约我市经济社会发展中的突出问题。坚持向管理要效益，转变观念、创新方式，着力加强城市建设、政府投资、公共财政、国有资产等各方面管理，切实完善制度规定，改进薄弱，堵塞漏洞，提高精细化、科学化管理水平，促进政府依法规范高效行政。

（三）更加注重发展经济与改善民生相促进。善政之要，惟在养民。坚持把民之所望作为施政所向，优先保障民生投入，优先安排民生项目，优先解决民生难题，让人民群众成为改革发展的最大受益者。深入实施“九项民生工程”，努力解决好就业、社保、教育、医疗、养老、住房等突出民生问题，着力打造宜居宜业环境，以惠民生凝结发展的共识，以优服务集聚人才和资源，推动创新创业创造。加强和创新社会管理，拓展联系群众的渠道，增进同人民群众的感情，促进社会公平正义，依法保障公民权益，让人民生活得更加幸福。

（四）更加注重市场作用与政府作为相结合。充分发挥市场在资源配置中的决定性作用，探索负面清单管理模式，推进资源要素配置市场化改革，市场能有效调节的经济活动取消行政审批，企业投资项目交由企业依法自主决策，依靠市场力量推进更好更快发展。更加有为有效地履行政府职能，以亮剑精神整治发展软环境，以过硬作风提升干部执行力，以务实态度推动工作新突破，努力把达州建成行政效能高、社会秩序好、发展环境优的魅力城市。

各位代表，达州人民勤劳智慧，不畏艰难，勇于进取。只要我们扭住发展不放松、面对困难不退缩、团结奋斗不懈怠，就一定能够克服各种困难，开启改革发展的崭新篇章！

三、2014年重点工作

做好今年政府工作，我们必须坚持整体推进，着力重点突破，促进提档升级。重点抓好以下五个方面的工作：

（一）全面深化改革开放，增强发展的动力和活力

深化经济体制改革。推进市县国有企业改革，完善现代企业制度，推动国有企业产权多元化发展，发展混合所有制经济。加快市罐头厂等4户停产企业改革，启动39户企业分类改革。推进金融改革，做强达州市城市商业银行，组建达州农村商业银行，引进2家以上银行来达设立分支机构，支持发展小额贷款公司、融资性担保公司等准金融机构。推进财税体制改革，完善政府预算管理体系，健全政府性债务风险防控机制，加强乡镇基本财力保障。推进投融资体制改革，规范地方政府融资平台管理，增强资本运营和风险管控能力。推进用地制度改革，加快建立城乡统一的建设用地市场。推进农村产权制度改革，开展农村土地确权颁证工作，积极筹建农村土地产权交易中心。推进国有林场改革，完善农田水利建设管护机制。积极争取国家天然气综合开发利用示范区，探索建立天然气资源开发补偿机制。

提高开放合作水平。坚持以开放促发展、促改革、促创新，加快构建开放型经济新体制，积极申报国家内陆开放试点城市。全面融入重庆发展，推动交通通道连接、产业配套发展、企业互动合作。大力开展“招商引资突破年”活动，积极推进产业链招商、园区招商、以商招商等招商模式，重点针对央企和国内外500强企业开展投资促进工作，着力创造条件促进达商“回家”，力争引进投资5亿元以上产业项目30个，招商引资到位资金550亿元。加快达州海关筹建步伐，支持达州经开区、大竹工业园区外向型产业基地建设，全年实现外贸进出口总额2.7亿美元。精心筹办第五届秦巴地区商品交易会，着力打造区域性有影响力的展会平台。

支持民营经济发展。坚持权利平等、机会平等、规则平等，完善促进民营经济发展的政策措施，充分激发民间投资热情，力争民间投资比重提高1个百分点以上。鼓励和支持民营企业抱团发展做大做强，支持达州商会大厦建设，展示达州民营企业新风貌。搭建中小微企业公共服务平台，设立共同基金，鼓励支持全民创业。新增私营企业1600户、个体工商户7000户以上。

（二）加快转方式调结构，推动经济持续较快增长

保持投资稳定增长。扎实开展“项目落实年”活动，继续实施重大项目带动战略，突出抓好25个重大工程项目，带动150个重点项目建设，确保完成全社会固定资产投资1150亿元。主动对接国家和省投资方向，切实加强项目前期工作经费投入，继续加大交通、水利、能源等基础设施投资力度，有效增加产业投资特别是工业、服务业投资比重，争取多上一批城市基础设施、棚户区改造、信息基础设施、节能环保产业等方面的项目。完善电力基础设施，加快“气化达州”步伐。加强和改进金融服务，争取发行第二期企业债券，扩大直接融资成效，新增信贷投放120亿元以上。加大土地整理力度，新增耕地2万亩以上。强化能源资源有效供给，建设中石油北外环至经开区输气管线。

推进工业转型升级。深入实施工业强市核心战略，确保规模以上工业增加值增长12.5%。积极推进天然气综合利用由化肥向精细化工升级换代，促进园区企业成链互补、循环发展，加快建设瓮福蓝剑磷酸盐、玖源三聚氰胺等项目，开工建设达兴能源D-III等项目，集中打造百亿化工产业集群。改造提升冶金、建材等传统产业，加快达钢搬迁改造步伐，推进钒钛钢铁产业基地建设。大力培育先进制造、节能环保装备、新材料等战略性新兴产业，引进发展清洁能源汽车制造业，开工建设安山岩纤维等项目。继续推进煤炭企业兼并重组，全面完成18户煤炭企业整顿关闭，实现结构、产能“双提升”。切实增强产业园区承载功能，完成“七通一平”1万亩，建成标准厂房35万平方米，力争达州经开区创建为国家经济技术开发区。大力实施企业“百十一”工程，新培育超10亿元企业2户、超亿元企业18户，新增规模以上工业企业60户。组织实施工业技改项目150项，完成投资265亿元。创建市级以上企业技术中心8家，培育省级以上创新型企业10家，申请专利600件。推进工业化信息化深度融合，培育信息化示范企业20家。探索发展物联网事业，加快智慧城市建设。

提升服务业发展水平。全面落实服务业发展支持政策，推进服务业发展提速、比重提高、水平提升，确保服务业增加值增长11%。继续抓好达州公路物流港、经开区铁路专用线等项目建设，推进火车货站南迁，全面启动秦巴物流园区基础设施建设，着力打造具有区域竞争力的现代物流园区。加快建设红星美凯龙、仁和春天、罗浮新城市广场等城市综合体，启动建设马踏洞金融集聚区，改造提升西城达人街、南城阳光耍都等餐饮特色示范街区。充分发挥旅游资源优势，完善要素配置，提升打造4A级景区，推进精品线路整合，创建旅游标准化示范市，力争实现旅游总收入90亿元。加快发展新型商业业态和新型消费模式，大力发展电子商务、网购网销，依法整顿和规范市场秩序，促进消费持续稳定增长。

加快农业现代化进程。深入开展粮油高产高效创建活动，抓好粮食生产重点县建设，力争粮油生产再获丰收。大力实施农业“4+8”品牌工程，新发展特色产业基地15万亩，新建畜牧养殖小区200个，创建“三品一标”认证品牌22个。加大涉农资金整合力度，加强农业综合生产能力建设，7个县（市、区）各建成3万亩以上集中连片农建综合示范区。推进农产品精深加工，加快市农产品加工集中区建设，新开工农产品加工项目15个。扶持发展种养大户、家庭农场等新型经营主体，新增市级以上龙头企业10家、标准化农民专业合作社20家，培训新型农民140万人次。强化农业科技服务和技术推广，主要农作物耕种收机械化水平达到35%。全面落实各项强农惠农富农政策，大力实施科普惠农兴村计划，落实农民增收书记、县（市、区）长负责制，促进农村群众致富奔小康。

（三）大力推进新型城镇化，促进城乡统筹发展

推进新型城镇化。坚持全域规划，注重多规衔接，科学制定达州新型城镇化中长期发展规划，加速构建“四层架构”城镇体系。大力推进产业新城和城市新区建设，加快发展一批工业重镇、商贸强镇和旅游名镇，力争新增城镇建成区面积16平方公里、人口12万，城镇化率提高1.8个百分点以上。稳步推进行政区划调整，积极争取撤县设区（市），启动实施一批符合条件的乡镇撤乡设镇、撤镇设街道办事处工作，开展扩权强镇改革试点。探索建立多元可持续的资金保障机制，做大做强服务新型城镇化的市级投融资平台。加快住房保障体系建设，开工建设保障性住房5040套，启动棚户区（危旧房）改造17830套（户）。实施“农民工住房保障行动”，提供农民工公共租赁住房1170套（间）。加快推进户籍制度改革，全面放开落户限制，稳步推进常住人口基本公共服务均等化，让进城人员更好地融入城市生活。

实施中心城区带动战略。围绕建设百万人口区域中心城市目标，拉大城市框架，优化空间布局，加强建筑形态、重要节点景观规划设计，完成市域和中心城区综合交通运输体系规划编制，提升城市功能和形象。加快北城滨江新区、南城三里坪新区、西城马踏洞和莲花湖片区建设，启动经开区长田坝新区建设，推进以塔坨片区为重点的旧城改造。抓好凤凰山隧道、环城路二期、金南大桥等道路桥梁建设，构建城区交通微循环，缓解城市拥堵。继续实施城市公交优先战略，优化公交线路设置和站点布局，加快公交首末站场建设，规范出租车运营管理，严厉打击非法违规营运。深化城乡环境综合整治，持续加大绿化美化、清扫保洁、治尘治噪、风貌塑造工作力度，突出整治农贸市场及火车站场周边环境，规范城市供水、排水、供电、供气等管线建设，积极创建国家卫生城市。建立完善城市管理长效机制，推进数字城管建设，提升城市管理水平。

统筹城乡区域发展。加大城乡协同发展力度，推进公共财政向农村倾斜、基础设施向农村延伸，促进以城带乡、城乡一体发展。围绕构建区域综合交通枢纽，加快巴达铁路、南大梁高速公路、达宣达开快速通道建设，力争开工达营、巴万高速公路，实施渠江航道升级改造，做好达州机场迁建、达渝达万城际铁路等项目前期工作，改造提升国省干线公路100公里，建成农村公路2120公里。加强农田水利设施建设，推进渠江流域综合整治，加快白岩滩等3座中型水库建设进度，开工建设石峡子水库，完成病险水库除险加固20座，新增有效灌面6万亩，新解决20万农村人口饮水安全问题。统筹推进新村建设“1+5”工程，建成幸福美丽新村45个，创建美丽乡村示范村庄52个，完成农房建设2.95万户，让越来越多农村群众过上现代文明生活。

加大扶贫开发力度。坚持把提高贫困人口生活水平和减少贫困人口数量作为主攻方向，落实目标、任务、资金和权责“四到县”制度，增强扶贫攻坚的精准性、有效性、持续性。大力推进基础扶贫、新村扶贫、产业扶贫、能力扶贫和生态扶贫“五大扶贫工程”，突出抓好连片特困地区基础设施和生态项目建设，加快资源开发和特色优势产业培育，增强发展造血功能。加快实施秦巴山区连片扶贫开发项目7个、整村推进项目45个，确保10万扶贫对象脱贫，尽快让贫困地区富起来、贫困群众生活好起来。

（四）切实加强环境治理保护，推进生态文明建设 推进节能减排。

强化节能降耗，严格限制新上高能耗、高污染项目，淘汰8户落后产能企业，确保万元生产总值能耗下降3.5%。严格执行环境标准，加强工业企业、城市扬尘污染治理，推进重点行业脱硫、脱硝、除尘改造，有效减少雾霾污染。全面推进清洁生产，力争达州经开区创建为国家循环化改造示范园区。综合治理重点流域水污染，加强饮用水源保护，启动第二饮用水源地选址工作。加快城镇垃圾、污水处理设施及配套管网建设，力争开工建设中心城区第二污水处理厂。加强农村面源、畜禽养殖污染治理。强化环境影响评价和环境监管，加大环境污染治理和惩处力度，让青山绿水长留、蓝天白云常驻。

抓好生态建设。深入推进天然林保护和自然保护区、生态功能区建设，启动新一轮退耕还林工程，完成营造林18万亩，森林覆盖率达到41.5%。抓好城市干道、城市周边和沿路、沿河、沿湖绿化美化，启动大寨子公园二期建设，新增城镇绿地38万平方米。严守耕地林地红线，加强建设用地预审管理，建立产业园区土地利用绩效评估制度。加大水土保持力度，综合治理水土流失面积100平方公里。强化重点区域地质灾害防治，抓好生态脆弱和灾毁区域生态修复，保障人民群众生命财产安全。

（五）努力保障和改善民生，维护社会和谐稳定

扎实办好民生实事。切实加大民生投入力度，确保公共财政用于民生支出比例不低于70%。坚持把就业作为民生之本，切实抓好高校毕业生、城镇困难人员、退役士兵就业创业工作，全年新增城镇就业3.5万人以上。深化收入分配制度改革，适时调整最低工资标准，推进企业工资集体协商。完善社会保障体系，推进城乡居民养老、医疗保险制度并轨，稳步提高城乡低保和“五保”供养水平。加强助残、救孤、济困和法律援助等惠民帮扶工作，加大困难群体重特大疾病医疗救助力度，资助4.7万名重度残疾人护理费用。推进社会养老服务体系建设，设立社区养老中心，探索居家养老新模式。健全社会福利制度，支持发展慈善事业。高度重视农村“留守”问题，保障妇女儿童合法权益。

大力发展社会事业。坚持立德树人导向，深化教育综合改革，提高教育教学质量。优化城乡教育资源配置，推进义务教育均衡发展，新改扩建中小学校121所、幼儿园14所。大力发展职业教育，加快西南职教园区建设。积极发展高中教育，推进高等教育、继续教育、特殊教育改革发展。大力引进省内外名校来达办学，积极利用信息化手段扩大优质教育覆盖范围。加快市中心医院业务综合楼、市妇幼保健院迁建工程、市食品药品及农产品综合质量检验检测中心建设，推进乡镇卫生院、村卫生室标准化改造。持续深化医药卫生体制改革，重点推进公立医院改革。加强重大传染疾病预防控制。大力发展中医药事业。广泛开展全民健身运动，做好省第十二届运动会和第八届残运会参赛组织工作。促进人口均衡发展，贯彻执行“单独二孩”政策。抓好第三次全国经济普查工作。大力发展哲学社会科学事业，加强民族、宗教、对台、外事、侨务、统计、档案、保密、气象、地方志等工作。

加快建设文化强市。深化文化体制改革，加快国有经营性文化单位转企改制。完善公共文化体系，实施文化惠民工程，完成市图书馆、城市规划馆、工青妇活动中心建设，加快莲花湖展演基地大剧院、文化馆及田园大舞台建设，提高达州博物馆陈展水平，抓好“乡镇（社区）文化院坝”建设。广泛开展群众性文化创建活动，办好第四届全国新农村文化艺术展演和首届省农民艺术节。加强文化遗产传承保护，启动宣汉罗家坝巴人文化遗址、渠县城坝遗址开发前期工作。抓好重点文化产业项目建设，支持非公有制文化企业和小微文化企业发展，力争文化产业增加值达到35亿元。

切实加强社会治理。探索创新社会治理体制机制，推进以信息化为支撑的网格化服务管理。深入开展普法教育，推进依法治市进程。完善“大调解”工作体系，有效化解社会矛盾，推行网上受理信访制度。强化社会稳定风险评估，加强社会治安专项整治，创新流动人口、特殊人群服务管理，不断深化“平安达州”建设。深入开展“扫黄打非”，切实加强网络监管。夯实安全生产基层基础，突出抓好道路安保设施建设，推动安全生产形势持续好转。深化食品药品安全专项整治，加强农产品质量安全监管，切实保卫“舌尖上的安全”。完善政府应急管理平台，着力提升突发事件应急处置能力。

大力支持驻达解放军和武警部队建设，切实加强国防教育、国防动员、人民防空等工作，推进国防后备力量建设。深入开展拥军优属工作，落实优抚安置政策，推动军民融合发展。

各位代表，我们的政府是人民的政府，让全市人民过上美好生活是我们的不懈追求。我们一定自觉肩负起转型升级、改革发展的历史使命，自觉担当起为民谋利、为民解忧的重要责任，创造无愧于历史、无愧于时代、无愧于人民的新业绩！

四、加强政府自身建设

面对艰巨繁重的工作任务，我们将以自我革命的精神，积极迎接深化改革和转型发展的大考，提振精气神，汇聚正能量，努力建设人民满意政府。

（一）加快转变政府职能。完成政府机构改革，推进事业单位分类改革。深化行政审批制度改革，实质性减少行政审批事项。清理调整非行政许可事项，规范中介服务行为。推进社会信用体系建设，建立失信惩戒机制。完善政务服务中心标准化建设，全面落实“两集中、两到位”，建立完善并联审批、网上审批和全程代办服务机制，切实提高行政效能。

（二）深入推进依法行政。坚持用法治思维和法治方式履行职能，建立行政权力清单制度，推进行政权力依法规范公开运行。严格行政决策程序，健全规范性文件和重大行政决策合法性审查制度。深化行政执法体制改革，减少行政执法层级，规范自由裁量权，加强行政复议和行政调解。自觉接受市人大及其常委会的法律监督、工作监督和市政协的民主监督，认真听取各民主党派、工商联、无党派人士和人民团体的意见，重视司法、舆论和公众监督。

（三）努力改进工作作风。扎实开展群众路线教育实践活动，严格执行“改进工作作风密切联系群众”有关规定，深入整治“四风”突出问题。有效治理“文山会海”，从严控制政府发文数量和会议数量。厉行节约、反对浪费，削减“三公”经费支出10%，严禁新建楼堂馆所。建立健全干部作风日常监督和考核评价机制，推进政府系统作风建设制度化常态化。

（四）切实加强廉政建设。严格执行党风廉政建设责任制，健全反腐倡廉制度体系。深化政府信息公开，建立电子政务大厅。开展政风行风满意度测评，推进政风行风持续好转。严格财务预算、核准和审计，加强工程建设、公共资源交易等重点领域监督，大力整治吃拿卡要等行为。保持惩治腐败的高压态势，支持执纪执法机关依法查办案件，努力建设廉洁政府。

各位代表，时代赋予重任，奋斗铸就辉煌。改革开放创新，我们已迈开大步；扩量提质增效，我们正奋力前行。让我们紧密团结在以习近平同志为总书记的党中央周围，在省委、省政府和市委的坚强领导下，团结一心、锐意进取，开拓创新、扎实工作，为加快建设川渝鄂陕结合部区域中心城市和川东北经济区核心增长极而努力奋斗！
